# High structural stability, reduced lattice-thermal conductivity, and elevated energy harvesting efficiency in a Lu_2_CoCrO_6_

**DOI:** 10.1039/d5ra07005h

**Published:** 2025-11-24

**Authors:** Samia Shahzadi, A. Elfasakhany, S. Nazir

**Affiliations:** a Department of Physics, University of Sargodha 40100 Sargodha Pakistan safdar.nazir@uos.edu.pk +92-334-9719060; b Mechanical Engineering Department, College of Engineering, Taif University Taif 21944 Saudi Arabia

## Abstract

Double perovskite oxides have been emerged as promising candidates for the fast evolving technical frontier, playing a key role in the development of efficient energy conversion devices to address global energy challenges. Therefore, we theoretically examined the structural stabilities, thermoelectric, electronic, and magnetic aspects of the ordered Lu_2_CoCrO_6_ structure. The calculated negative formation enthalpy (−4.2 eV per atom), lack of imaginary modes in the phonon curves, and elastic constants that meet the Born conditions, confirms the thermodynamical, dynamical, and mechanical stability of the system, respectively. The material is classified as ductile by Pugh's ratio 
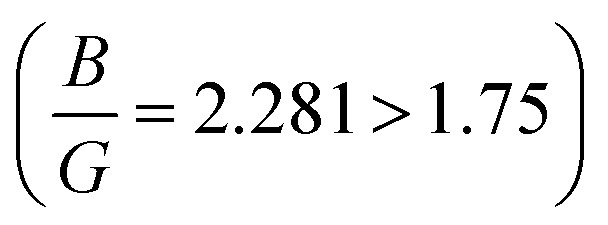
 and Poisson's ratio (*ν* = 0.308 > 0.26). 
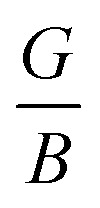
 ratio (0.438), along with a *ν* value, which affirms ionic bonding. Also, the material exhibits a semiconducting state having direct a band-gap of 1.13 eV. The antiferromagnetic superexchange coupling between Co^3+^ (3d^6^) and Cr^3+^ (3d^3^) ions *via* oxygen favors the ferrimagnetic stable state. Further, the calculated partial spin magnetic moment of 3.11/−2.52 *µ*_B_ on the Co/Cr ion, along with an isosurface plot of the spin magnetization density, further validates the ferrimagnetic phase of the material. Interestingly, thermoelectric study demonstrates that enhanced phonon scattering causes the lattice thermal conductivity (*k*l) to drop with increasing temperature, results in a giant figure-of-merit of 1.00 at *µ* = 0.1 Ry at 700 K. Hence, these results revealed that LCCO is stable and keeps multifunctional features that may be favorable for utilization in thermoelectric and spintronic devices.

## Abstract 2

Double perovskite oxides have been emerged as promising candidates for the fast evolving technical frontier, playing a key role in the development of efficient energy conversion devices to address global energy challenges.

## Graphical abstract

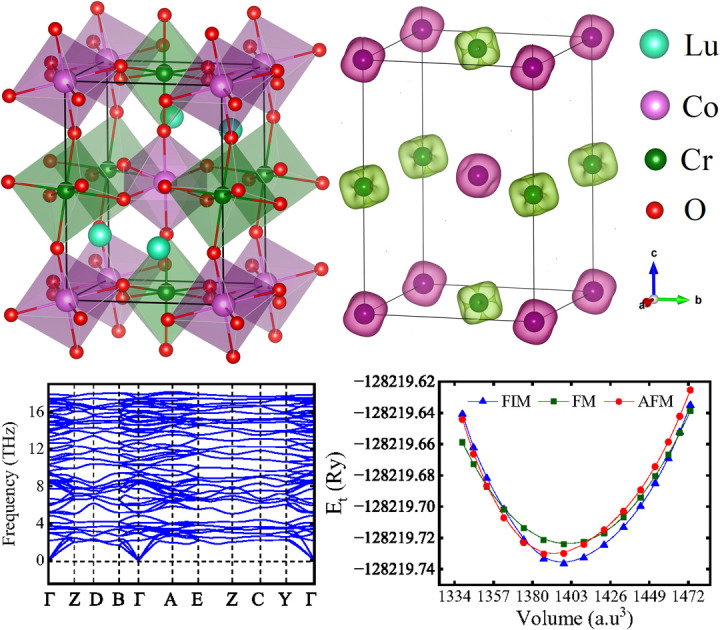

## 1 Introduction

The global shift towards advanced technology and sustainable energy has led to a growing need for materials that can meet the high-performance requirements of modern applications.^1–3^ Materials that exhibit strong electronic and magnetic aspects, while maintaining long-term stability under varying temperatures (temp.) and environmental conditions, are essential for applications in energy storage, spintronics, and environmental remediation.^4^ The traditional materials frequently fail to meet these requirements, which leads to the investigation of novel chemical groups with improved features. The transition metals (TM) and rare-earth-based double perovskite oxides (DPOs), having the general formula A_2_BB′O_6_, display significant structural diversity, making them hot candidates for a range of applications such as optical, electrical, magnetic, and catalytic uses.^5^ Due to their strong spin polarization, ferromagnetic (FM) phase stability, and excellent Curie temp. (*T*_C_), DPO has a number of appealing possibilities.^6^ Furthermore, these materials show remarkable features within their family, including insulating ferrimagnetic (FIM) states,^7^ magneto-dielectric features, magneto-optic aspects,^8^ half-metallic (HM) behavior,^9^ and multi-ferroic^10^ capabilities. Along with this, a number of DPOs have recently been discovered keeping interesting applications in the fields of photovoltaic devices,^11^ photocatalysis,^12^ and photoelectro chemical energy storage systems.^13^ Also due to the intriguing properties, including magnetodielectric effect,^14,15^ colossal magnetoresistance effect,^8^ multiferriocity behavior,^16^ La_2_CoMnO_6_ has emerged as the subject of much matter. Likewise, La_2_CoMnO_6_ and La_2_NiMnO_6_ represents the most extensively examined ordered DPOs, exhibiting FM semiconducting (SC) phase with *T*_C_ near room temp. (220 K for La_2_CoMnO_6_ and 280 K for La_2_NiMnO_6_).^17,18^ However, Nd_2_CoMnO_6_ has similarities with La_2_CoMnO_6_, yet it has received less attention in research. It exhibits FM and meta-magnetic features,^19^ spin-phonon coupling,^20,21^ MD coupling,^15^ phase separation, and polaronic hopping conduction^22^ along with multiferroic behavior.^23^

Particularly, A_2_FeCrO_6_ (A = Pr, Bi) DPOs with two 3d TM at the B and B′ sites demonstrate the intriguing optoelectronic aspects as they display significant absorbance in the visible range of solar radiation and a favorable band gap energy (*E*_g_).^24–26^ The FM or FIM behavior of the Cr-based ordered A_2_CrMO_6_ (A = Sr,Ca; M = Mo, W, Re) structures have also been reported, whereby Cr^3+^ (3d^3^, *S* = 3/2) and Mo^5+^ (4d^1^, *S* = 1/2), W^5+^
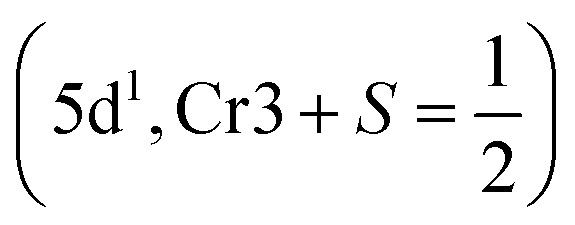
, Re^5+^ (5d^2^, *S* = 1) paired antiferromagnetically.^27^ The Sr-based compounds such as Sr_2_CrMO_6_ (M = Mo and W) have high *T*_C_ 473 and 453 K, respectively, according to their research, but the Ca-based Ca_2_CrMO_6_ (M = Mo/W) have a low *T*_C_ of around 148/143 K.

Recently, the growing demand for thermoelectric (TE) materials, capable of recovering and converting waste heat into electrical energy at elevated temp., has led to increased interest in DPOs. These materials are favored due to their low thermal conductivity (*k*), high thermal stability, and environmentally friendly aspects compared to IVA-VIA TE alloys.^28–30^ However, the TE features have been investigated through both experimental and theoretical approaches for a limited number of DPOS, including La_2_CoMnO_6_,^17,31,32^ La_2_NiMnO_6_, Sr_2_BB'O_6_,^33^ and Pr_2_CoFeO_6_,^34^ among others. As the figure of merit (*ZT*) is a dimensionless parameter used to assess a TE material's overall performance. There are two main ways to increase *ZT*, improving the Seebeck coefficient (*S*) by optimizing the material's electronic structure and reducing the overall *k*, especially by suppressing the lattice *k* (*k*_l_).^35,36^ The chemical composition of the material has a significant impact on the *k*_l_, predominantly by means of lattice softening effects and phonon scattering processes [10]. Furthermore, the total number of atoms in the primitive unit cell has an important impact on determining its magnitude.^37^ In this context, Mustafa *et al.*, investigated the halide DPOs and found that the *ZT* at 800 K for K_2_YAgBr_6_ and K_2_YAgI_6_ were high of 0.68 and 0.74, respectively.^38^

Motivated by the variety of applications of DPOs, a thorough analysis of the Lu_2_CoCrO_6_ (LCCO) has been performed, another possibility for multifunctional applications. To the best of our knowledge, its electronic, magnetic, and TE aspects have not yet been systematically explored, leaving a clear gap in the reported literature. Hence, examining LCCO allows us to understand how the presence of Co^3+^ and Cr^3+^ ions affects the superexchange mechanism and TE behavior, providing valuable comparative insight within the broader class of DPOs. Therefore, we comprehensively investigate its structural stability through formation enthalpy (Δ*H*_f_), mechanical, and dynamical behavior. The magnetic ordering is governed by superexchange interactions, together with electronic and magnetic aspects. To determine its potential in energy conversion technologies, we study its TE performance. The objective of this extensive investigation is to reveal the inherent physical features of the LCCO and provide the foundation for its implementation in TE and spintronic applications.

## 2 Computational and structural details

Spin-polarized (SP) density functional theory calculations were performed using the full potential linearized augmented plane wave (FP-LAPW) approach, which is implemented in the WIEN2k code.^39^ To effectively account for electron correlation effects, the generalized gradient approximation (GGA) is employed alongside an on-site Coulomb interaction (GGA + *U*), utilizing *U* value of 5.0/3.5 eV for Co/Cr-3d states.^40^ In the context of wavefunction expansion within atomic spheres, the angular momentum cutoff is established at *l*_max_ = 12. Additionally, the plane wave cutoff is specified by *R*_mt_ × *K*_max_ = 7 with *G*_max_ set to 24. The integration over the Brillouin zone is conducted by utilizing a 6 × 6 × 4 Monkhorst Pack k-mesh, resulting in 76 *k*-points within the irreducible wedge, thereby guaranteeing satisfactory convergence. Atomic positions are thoroughly optimized until the Hellmann–Feynman forces acting on all atoms decrease to below 5 mRy/a.u. The convergence criteria for achieving self-consistency are established at 10^−5^ Ry for total energy (*E*_t_) and charge density. The TE parameters are evaluated by applying Boltzmann transport theory under the constant relaxation time of 10^−14^ s approximation using the BoltzTraP code.^41^

**Fig. 1:**
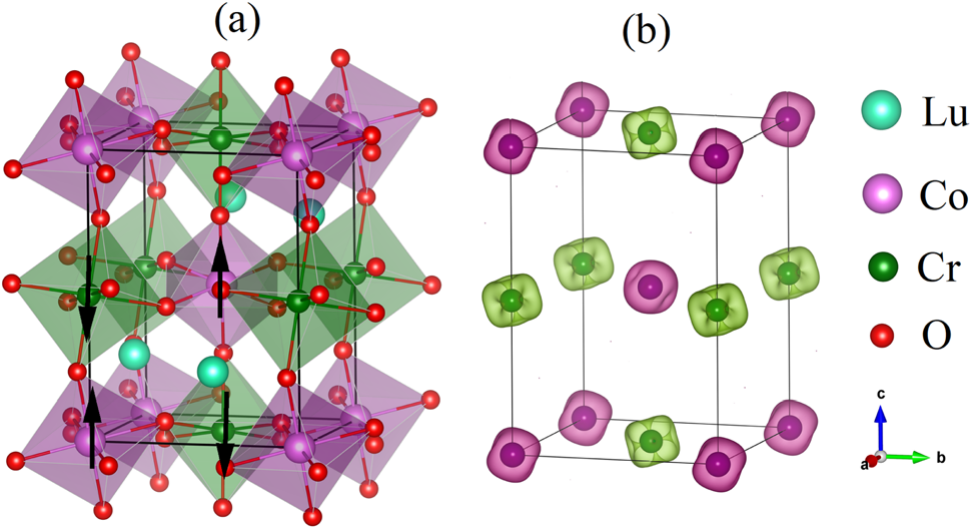
Schematic illustration of the (a) crystal diagram and (b) spin magnetization density plot in a ferrimegnetic spin ordering for the Lu_2_CoCrO_6_ structure.

The LCCO crystallizes in a monoclinic symmetry with the space group *P*2_1_/*n* (No. 14). The experimentally determined lattice parameters are *a* = 5.1416 Å, *b* = 5.4723 Å, *c* = 7.4158 Å, and *β* = 89.0211°.^42^ There are 4Lu, 2Co, 2Cr, and 12O atoms in its primitive unit cell. The atomic positions within the unit cell are defined as follows: Lu occupies the Wyckoff site 4e with coordinates (0.525, 0.571, 0.251); Co/Cr is located at 2d (0.5, 0, 0)/2c (0, 0.5, 0), respectively. The three distinct oxygen atoms O1, O2, and O3 occupy 2e positions at (0.391, 0.970, 0.248), (0.165, 0.154, −0.057), and (0.315, 0.683, −0.059), correspondingly.^42^ Experimentally, the distribution of Co and Cr ions at the B-site is found to be partially disordered. However, in the present study, we assume a perfectly ordered arrangement for computational simplicity. The LCCO crystal structure and spin magnetization density plots are depicted in Fig. 1(a) and (b), respectively.

## 3 Results and discussion

### 3.1 Structural stabilities

As the Goldschmidt tolerance factor (*τ*) is often employed to anticipate the crystallographic phase and evaluate the structural stability of the oxides, which is computed as:^43,44^1
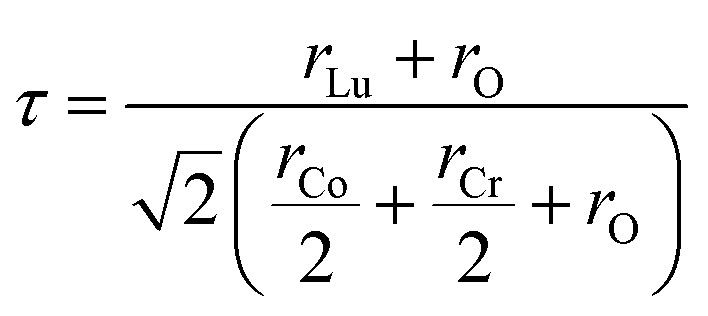
in which *r*_Lu_, *r*_Co_, *r*_Cr_, and *r*_O_ represent the ionic radii of these cations, having values of 1.03 Å, 0.55 Å, 0.62 Å, and 1.40 Å, respectively. As a result, *τ* ≈ 0.87, which falls within the standard stability range of 0.8 ≤ *τ* ≥ 1.0 and is significantly below 0.97, suggests a monoclinic DPO framework as opposed to an ideal cubic structure.^45^

Now, formation enthalpy (Δ*H*_f_) is calculated to analyze the thermodynamic stability of the structures as:2
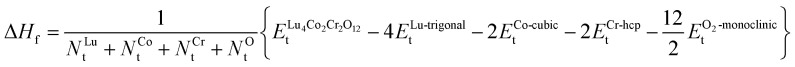
where 
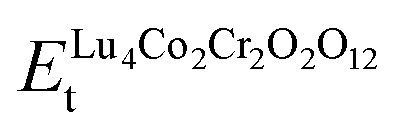
, *E*^Lu-trigonal^_t_, *E*^Co-cubic^_t_, *E*^Cr-cubic^_t_, and 
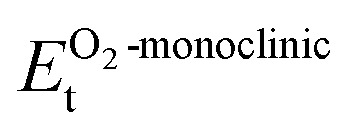
 represents the *E*_t_ of the LCCO motif, Lu (*R*3̄*m*-166), Co (*Fm*3̄*m*-225), Cr (*Fm*3̄*m*-225), and O (*C*2/*m*-12) molecule in their respective ground states, correspondingly. The computed Δ*H*_f_ is −4.22 eV per atom, where a negative sign confirms the thermodynamic stability of the structure.

Next, to evaluate the mechanical stability of the structure, we calculated the elastic tensors (*C*_*ij*_) through the application of six finite deformations and an analysis of the stress–strain response.^46,47^ In the monoclinic phase of the structure, we observe 13 independent *C*_*ij*_, which are in accordance with the necessary and sufficient Born criteria for the mechanical stability of the materials as follows:^48^*C*_11_ > 0, *C*_22_ > 0, *C*_33_ > 0, *C*_44_ > 0, *C*_55_ > 0, *C*_66_ > 0*C*_33_*C*_55_ – *C*_35_^2^ > 0, *C*_44_*C*_66_ – *C*_46_^2^ > 0, *C*_22_ + *C*_33_ – 2*C*_33_ > 0*C*_22_(*C*_33_*C*_55_ – *C*_35_^2^) + 2*C*_23_*C*_25_*C*_35_ – *C*_23_^2^*C*_55_ – *C*_25_^2^*C*_33_ > 02*C*_15_*C*_25_(*C*_33_*C*_12_ – *C*_13_*C*_23_) + *C*_15_*C*_35_(*C*_22_*C*_13_ – *C*_12_*C*_23_) + *C*_25_*C*_35_(*C*_11_*C*_23_ – *C*_12_*C*_13_) − *h* + *C*_55_*g*and*g* = *C*_11_*C*_22_*C*_33_ – *C*_11_*C*_23_^2^ + 2*C*_23_*C*_25_*C*_35_ – *C*_23_^2^*C*_35_ – *C*_25_^2^*C*_33_*h* = *C*_15_^2^(*C*_22_*C*_33_ – *C*_23_^2^) + *C*_25_^2^(*C*_11_*C*_33_ – *C*_13_^2^) + *C*_35_^2^(*C*_11_*C*_22_ – *C*_12_^2^) > 0

The calculated *C*_*ij*_ (see matrix below) for the LCCO DPOs satisfy the specifications given above inequalities, thereby affirming their mechanical stability.
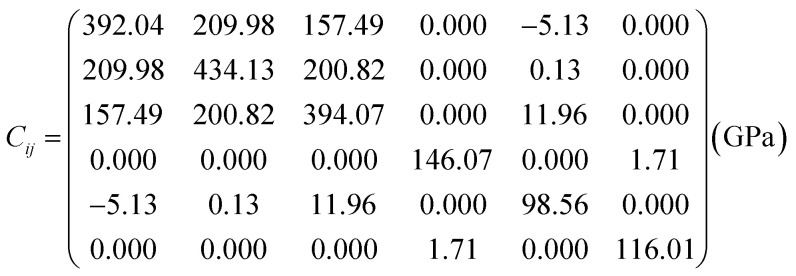


Furthermore, elastic constants play a significant role in examining the stiffness, brittleness, ductility, and anisotropy of the material using Bulk modulus (*B*)/Young's modulus (*Y*)/Shear modulus (*G*). The substantial hardness of the LCCO is indicated by its higher resistance to volume deformation under external pressure, as indicated by its B value of 260.36 GPa. *Y* = 298.66 GPa and *G* = 114.1 GPa validate its remarkable resistance to elastic deformation under uniaxial force, further demonstrating its intrinsic stiffness. Also, it is categorically classified as ductile by the Pugh's ratio 
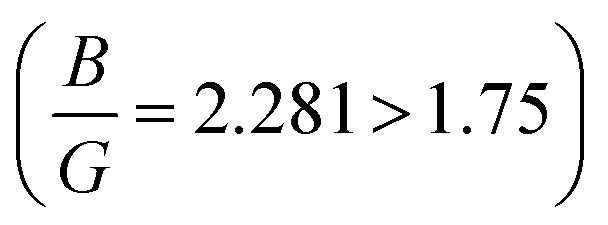
 and Poisson's ratio (*ν* = 0.308 > 0.26),^49^ both of which surpass the crucial criteria for brittleness. The *ν* and 
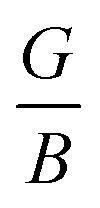
 ratio can be used to clarify the material's bonding nature. The 
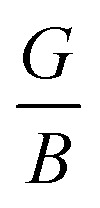
 ratio of 0.438 indicates the ionic nature of the system, because ionic materials usually have a value of 0.6, which is closer to ionic bonding. While *ν* = 0.25 implies ionic bonding, and *ν* = 0.1 hints at covalent bonding.^49–52^ The computed *ν* of 0.30881 shows a dominance of ionic bonding, because of its near to 0.25.

**Fig. 2:**
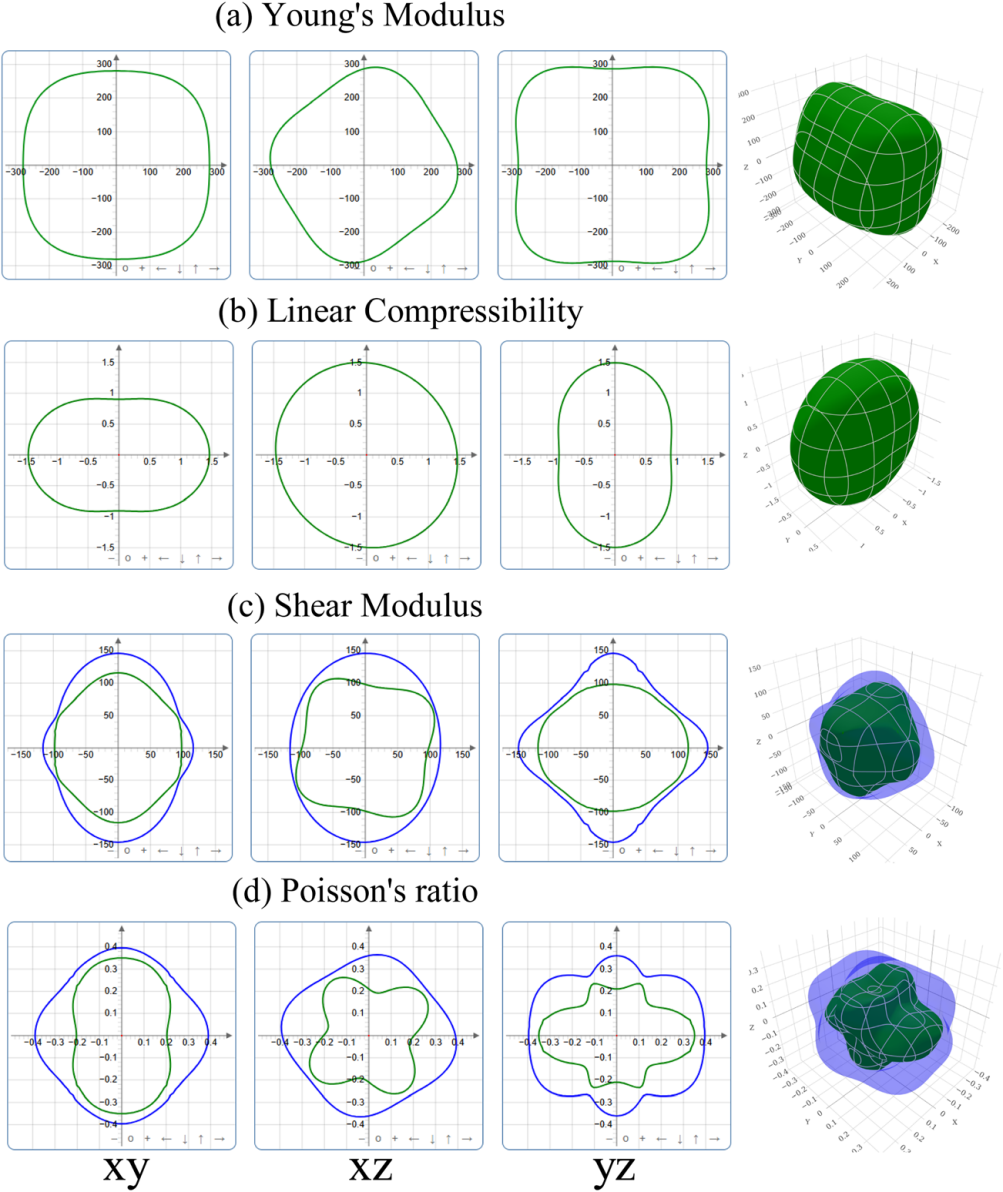
Calculated 3D-directional dependent elastic parameters for the Lu_2_CoCrO_6_ structure.

Likewise, Fig. 2 depicts the 2D and 3D anisotropic representations of the elastic parameters for the LCCO system along the *xy*, *xz*, and *yz* planes, and their corresponding calculated values are listed in Table 1. The plotted surfaces strong directionality illustrates the compound's mechanical anisotropy. For an ideal isotropic case, these mentioned surfaces are perfectly spherically symmetric; however, the degree of variation from a perfect sphere indicates the degree of elastic anisotropy, is closely linked to structural symmetry and bonding properties. As Y (see Fig. 2(a)) displays moderate anisotropy having value of 1.45, refers that stiffness varies noteably with crystallographic orientation. In contrast, Fig. 2(b)–(d) represents that *β*/*G*/*ν* shows a stronger directional dependence in contrast to *Y*, demonstrating substantial variation in compressibility, shear resistance, and lateral strain between various planes. These results highlight that LCCO is elastically anisotropic with significant orientation-dependent stiffness and compressibility. Moreover, the bonding nature of the material is explain in terms of charge density map as displayed in Fig. 4. It is clear that charge density is found to be strongly localized around the O atoms, while very little electron density is observed between the metal atoms (Co, Cr, and Lu). The absence of shared electron clouds between the metal–metal sites confirms that there is no direct covalent bonding among them. Instead, the bonding is mainly ionic in character, with oxygen acting as the electron-rich center coordinating the metal cations. This supports and strengthens the conclusion drawn from *ν*.

**Table 1:** Calculated Young's modulus (*Y*_min ._/*Y*_max ._) in GPa, linear compressibility (*β*_min ._/*β*_max ._) in TPa^−1^, shear modulus (*G*_min ._/*G*_max ._) in GPa, Poisson's ratio (*ν*_min._/*ν*_max._), and the corresponding anisotropic factor (A) for the Lu_2_CoCrO_6_ structure

*Y*_min ._	*Y*_max ._	*A*	*β*_min ._	*β*_max ._	*A*	*G*_min ._	*G*_max ._	*A*	*ν*_min ._	*ν*_max ._	*A*
244.12	353.92	1.45	0.90	1.53	1.69	95.28	146.17	1.53	0.186	0.426	2.28

**Fig. 3:**
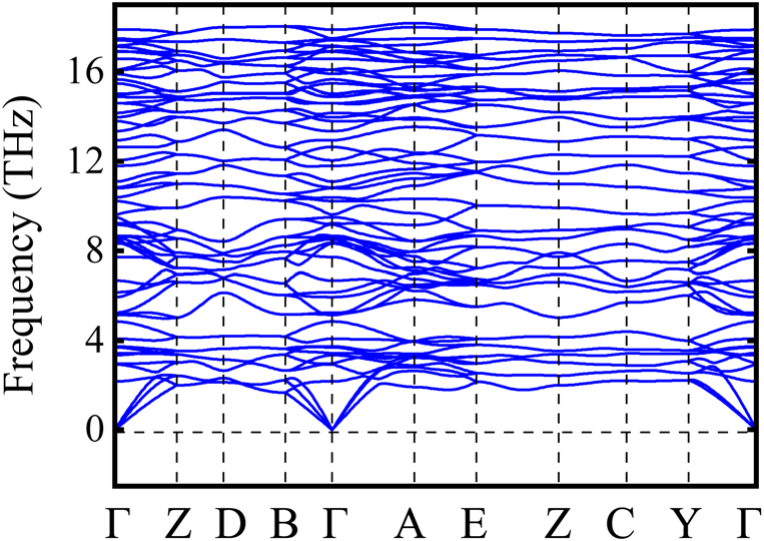
Calculated phonon spectrum of the Lu_2_CoCrO_6_ structure.

Now, the phonon dispersion curve along the high-symmetry route (Γ Z D B Γ A E Z C Y Γ) is computed in Fig. 3 to access the dynamic stability of the LCCO system. Hence, the lack of imaginary phonon frequencies across the Brillouin zone confirms that the LCCO motif is dynamically stable.^53,54^ In principle, the number of atoms in the primitive unit cell determines the number of phonon branches. Each atom contributes three vibrational modes having a total of 3*n* branches, where *n* is the number of atoms. These consist of 3*n* − 3 optical and 3 acoustic modes.^55^ There are 20 atoms in the primitive unit cell, the phonon dispersion shows 60 branches with 57 optical and 3 acoustic. Acoustic modes are represented by the lower frequency portion of the dispersion curves, whereas optical modes are represented by the higher frequencies. Oxygen atoms are the source of the high frequency optical modes because of their low atomic mass, which is inversely correlated with vibrational frequency. On the other hand, heavier atoms like Lu, Co, and Cr provide a larger contribution to the low frequency acoustic modes.

Finally, the quasi-harmonic Debye model^56,57^ has been used to calculate the thermodynamic aspects in the temp. ranges of 0 to 1000 K at a constant pressure of 0/5/15/25 GPa. Fig. 5 displays the variation of volume (V), Debye temp. (*θ*_D_), thermal expansion coefficient (*α*), entropy (S), specific heat capacity at constant volume (*C*_V_), and the specific heat capacity at constant pressure (*C*_P_). Due to compressive forces and thermal expansion, the unit cell volume decreases with pressure and increases with temp. as presented in Fig. 5(a).

**Fig. 4:**
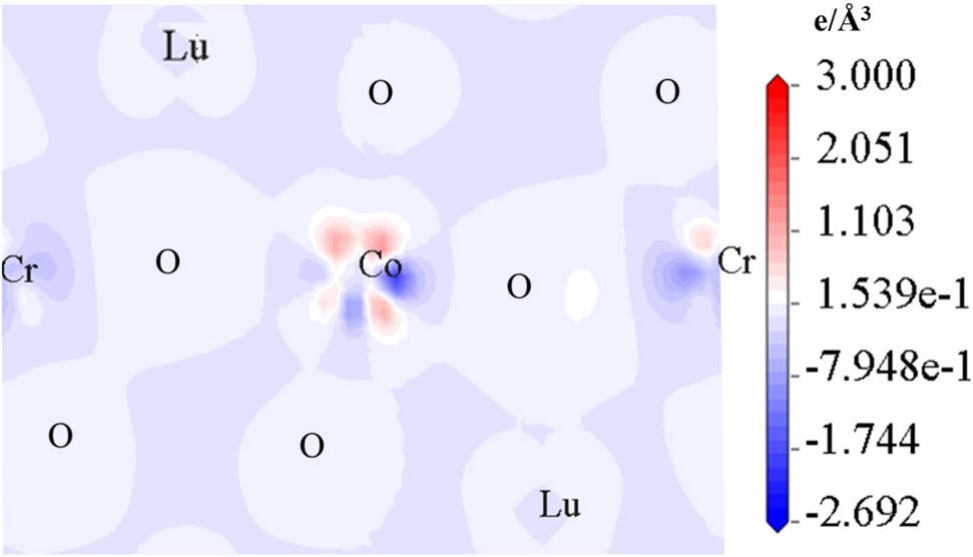
Computed charge density plots of the Lu_2_CoCrO_6_ structure.

**Fig. 5:**
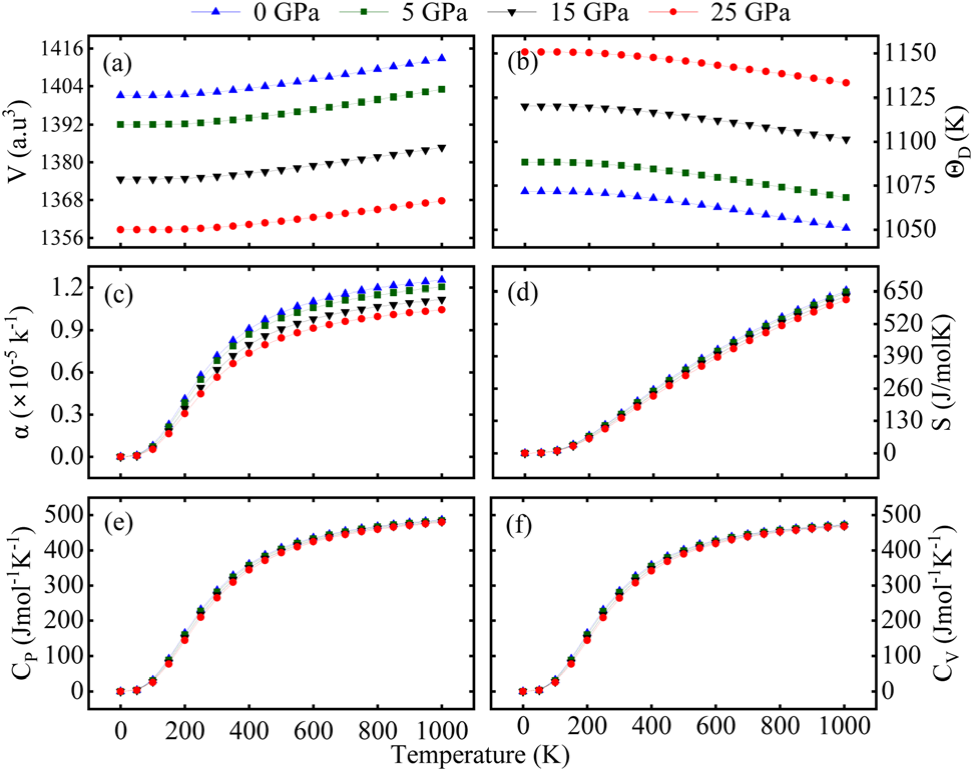
Computed (a) volume (V), (b) Debye temperature (*θ*_D_), (c) absorption coefficient (*α*), (d) entropy (S), and (e) and (f) specific heat capacity at constant pressure/constant (*C*_P_/*C*_V_) volume of the Lu_2_CoCrO_6_ structure.

Along with this, Fig. 5(b) shows that *θ*_D_ decreases as temp. rises, a phenomenon linked to the softening of phonon modes at higher temp. On the other hand, when pressure increases, *θ*_D_ rises, indicating the lattice stiffens, resulting in a less interatomic space. Now, Fig. 5(c) displays how the temp. and pressure affects the *α*. Up to 300 K, a sharp increase in its value is observed with temp. and a slow or near-saturation point appears beyond 700 K. It shows that pressure has an inverse relation with *α*: as pressure rises, *α* falls rapidly. This reduction is attributed to the suppression of atomic displacements under compression, which limits the value of *α*. This finding demonstrates the material's thermal resilience under various thermodynamic circumstances and provides important information about its volumetric response to temp. Fig. 5(d) displays S, commonly referred to as disorder in systems. It demonstrates the variance of entropy in connection with particular pressures and temp. An increase in lattice vibration brought on by a rise in temp. may excite electrons, increasing the system's entropy further. Fig. 5(e) and (f) illustrates how *C*_P_/*C*_V_ varies with respect to temp. and pressure, which serve as important indicators of phase stability and lattice dynamics. As can be seen, in the low-temp. region (0–400 K), *C*_V_*/C*_P_ grows dramatically with temp., indicating the quick triggering of phonon modes. At temp. over 400 K, the rise in *C*_V_ slows and approaches the conventional Dulong–Petit limit at 700 K, confirming harmonic behavior at high temp. Both *C*_P_ and *C*_V_ exhibit a modest drop in magnitude at any given temp. as pressure increases, which is explained by suppressed lattice vibrations brought on by volume contraction.

### 3.2 Magnetic aspects

**Fig. 6:**
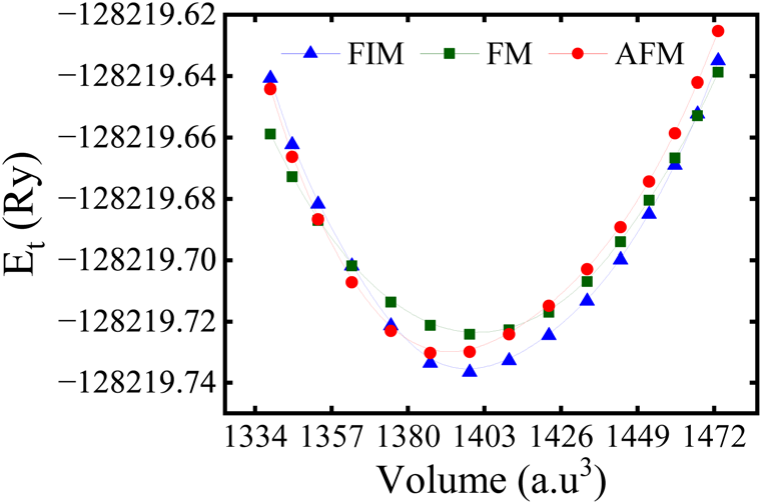
Calculated optimized energy-volume relationship for the Lu_2_CoCrO_6_ structure in the ferrimagnetic (FIM), ferromagnetic (FM), and antiferromagnetic (AFM) spin ordering.

To study the magnetic ground state, the structure was initially optimized by considering three spin-orderings (SO), such as FM, FIM, and antiferromagnetic (AFM) to precisely ascertain the equilibrium lattice parameters, volume, and *E*_t_ at the ground state using the Murnaghan equation of state. The *E*_t_ was computed as a function of volume for all three considered configurations as presented in Fig. 6, which confirms that the system is more stable in the FIM SO. The Co and Cr ions are anti-aligned (↑↓) in the FIM SO and remained aligned (↑↑) in the FM ones, and the individual ions of Co/Cr are aligned antiparallel (↑↓)/(↑↓) in AFM SO. The energy differences were calculated as Δ*E*_1_ = *E*_FIM_ – *E*_AFM_ = −31.54 meV and Δ*E*_2_ = *E*_FIM_ – *E*_FM_ = −186.56 meV. The negative signs indicate that the FIM SO is more stable than that of the FM and AFM ones, which is in agreement with the recent experimental findings.^42^

To further examine the magnetic behavior of the system, the total/partial spin magnetic moment (*m*_t_/*m*_s_) is computed. The estimated *m*_t_ is 2.00 *µ*_B_/f.u. with the *m*_s_ on the Co/Cr is 3.11/−2.52 *µ*_B_. The “–” sign on the Cr *m*_s_ signifies that the magnetic moments of Co and Cr are oriented antiparallel (↑↓) to one another, thereby affirming the existence of AFM coupling, which results in a net FIM SO within the system. Additionally, the computed *m*_s_ of the Co/Cr ion indicates that they lie in a +3(t^3^_2g_↑t^1^_2g_↓e^2^_g_↑e^0^_g_↓)/+3(t^3^_2g_↑t^0^_2g_↓e^0^_g_↑e^0^_g_↓) electronic configuration. Similarly, the computed orbital moment (*m*_l_) for the Co/Cr is 0.103/0.042 due to the SOC effect. Further, the 3D spin magnetization density of the system is illustrated in Fig. 1(b), clearly demonstrating the AFM coupling between Co and Cr ions. The distinct colors of the isosurfaces validate their AFM coupling and the slightly more spatial distribution around the Co-site indicates its larger *m*_s_ value in comparison to that of Cr. The observed asymmetry in the spin density magnitude corresponds with the calculated *m*_s_, highlighting the significant influence of the Co on the magnetic behavior of the compound. Further, the distinct separation of spin densities provides additional evidence for the AFM coupling, aligning with the anticipated orbital interactions in this DPO.

**Fig. 7:**
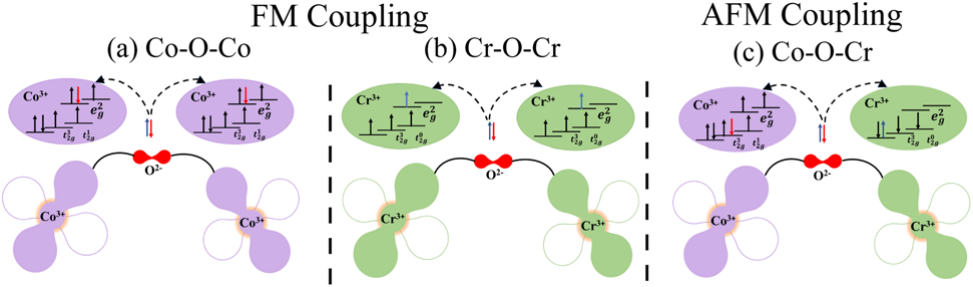
Schematic illustration of the ferromagnetic (FM) superexchange interactions of (a and b) Co–O–Co/Cr–O–Cr and (c) antiferromagnetic (AFM) coupling between Co^+3^ 3d^6^ and Cr^+3^ 3d^+3^ ions *via* oxygen in the Lu_2_CoCrO_6_ structure.

For more understanding about the interactions, the various forms of superexchange interactions between magnetic ions mediated by oxygen 2p orbitals are depicted in Fig. 7. The Co(e^2^_g_↑)–O–Co(e^2^_g_↑)/Cr(e^0^_g_)–O–Cr(e^0^_g_) interactions demonstrate FM coupling, characterized by the alignment of the Co/Cr *m*_s_ in a ↑↑/↑↑*o*rientation as shown in Fig. 7(a) and (b). Coversely, the charge transfer takes place between the partially filled Co^3+^–t^1^_2g_↑ and Cr^3+^–t^0^_2g_↑ orbitals, where electrons hop back and forth across the intermediate O^2−^ 2p states. In this way, a FIM ground state is stabilized by this indirect exchange process, which takes the form of an AFM superexchange *via* the Co–O–Cr route (see Fig. 7(c)).

### 3.3 Electronic structure

**Fig. 8:**
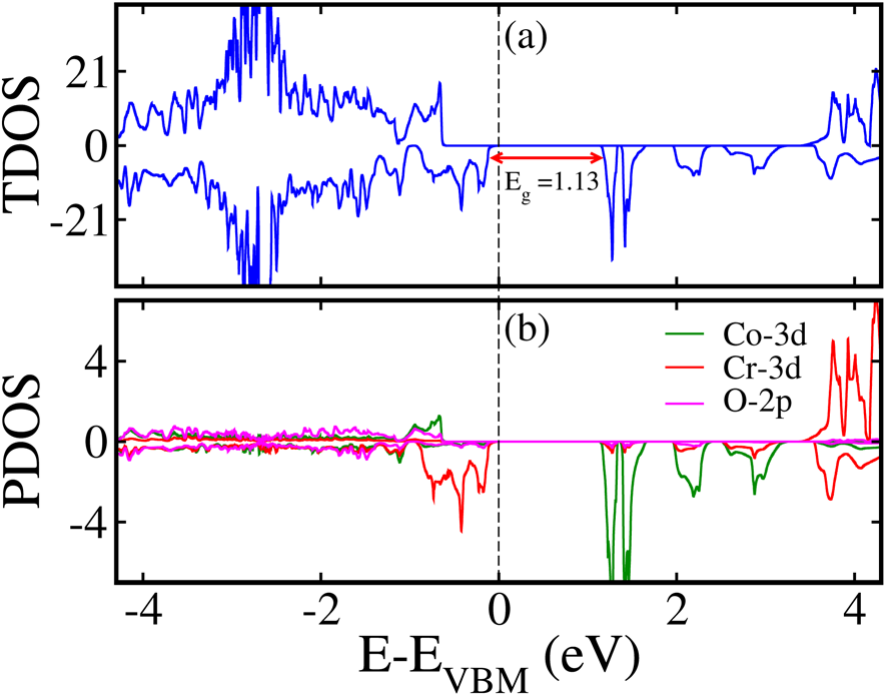
Calculated spin-polarized (a) total density of states (TDOS) and (b) partial density of states (PDOS), highlighting the role of Co-3d/Cr-3d/O-2p orbitals around the Fermi level for the Lu_2_CoCrO_6_ structure.

**Fig. 9:**
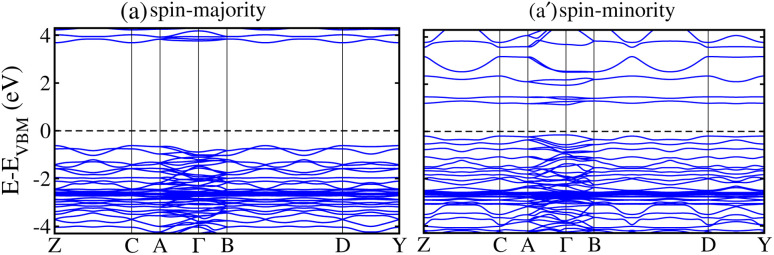
Calculated spin-polarized electronic band structures of the (a) spin-majority/(a′) spin-minority channel for the Lu_2_CoCrO_6_ structure.

To elucidate the electronic structure of the LCCO motif, the computed SP total density of states (TDOS) and the atom-resolved partial density of states (PDOS) in the FIM SO *via* the GGA + *U* method are depicted in Fig. 8(a) and (b), correspondingly. Fig. 8(a) illustrates that the system is a SC with an *E*_g_ of 1.13/3.96 eV in the spin-minority/spin-majority channel (N↓)/(N↑). Hence, the material real *E*_g_ is 1.13 eV, which further affirms its SC nature.To acquire a deeper understanding of the states adjacent to the Fermi energy (*E*_F_), we plotted the Co/Cr-3d and O-2p orbital resolved PDOS in Fig. 8(b). A significant contribution close to (*E*_F_) at the edge of the valence band (VB) arises from the Cr-3d orbitals. In a similar manner, the edge of the conduction band (CB) is primarily characterized by significant hybridization between the 3d orbitals of the Cr, along with minor contributions from Co-3d states (see Fig. 8(b)). Additionally, the calculated SP band structures are presented in Fig. 9 along with the high symmetry directions of the monoclinic Brillouin zone, further displaying the SC behavior of the material. A direct *E*_g_ of 1.13/3.96 eV in the N↓/N↑ exists and corroborates the computed TDOS in Fig. 8(a). Furthermore, we plotted the GGA + *U* + SOC calculated TDOS/PDOS in Fig. 1S of the supporting information (SI) in the stable FIM SO. Our results indicate that SOC does not significantly affect the electronic structure of the material; however, a small enhancement of 0.08 eV in the *E*_g_ with the inclusion of SOC is revealed. Analysis of the PDOS hints that the primary contributions to the TDOS near the *E*_F_ are attributed to Cr-3d orbitals (see Fig. 1S(b) of the SI). Moreover, here we would like to mentioned that why we adopted *P*2_1_/*n* structure of the LCCO, which corresponds to the ordered Co–Cr arrangement. Although the nominal valences reported there were Co^2+^/Cr^4+^, our DFT results yield Co^3+^ and Cr^3+^. This charge state is consistent with the *m*_s_ balance in the system: Co^3+^ and Cr^3+^ provide the correct total spin configuration and stabilize the experimentally consistent magnetic ordering. Thus, the *P*2_1_/*n* structure is retained, while electron redistribution leads to a Co^3+^/Cr^3+^ state that achieves magnetic and electronic stability.

### 3.4 Thermoelectric features

Finally, to study the TE aspects, first *κ*_l_ has been evaluated utilizing Slack's equation as:^58^3
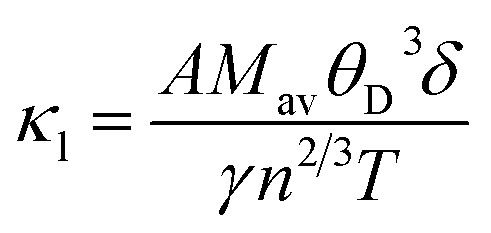
In this context, *M*_av_ represents the average atomic mass of the crystal, *δ* denotes the cube root of the average atomic volume, *n* indicates the number of atoms in the unit cell, *T* refers to the absolute temp., *γ* signifies the Grüneisen parameter, derived *ν* through the following equation:4
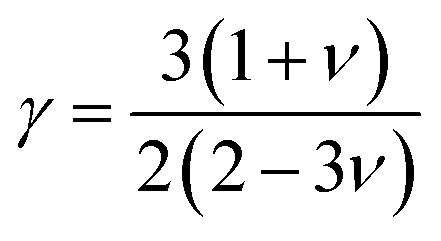
The factor *A* and *θ*_D_ are determined as:^59,60^5
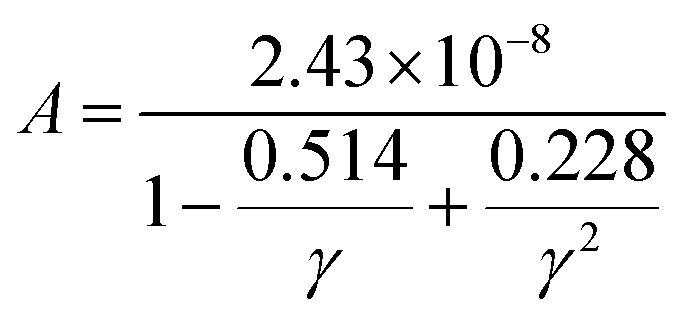
6
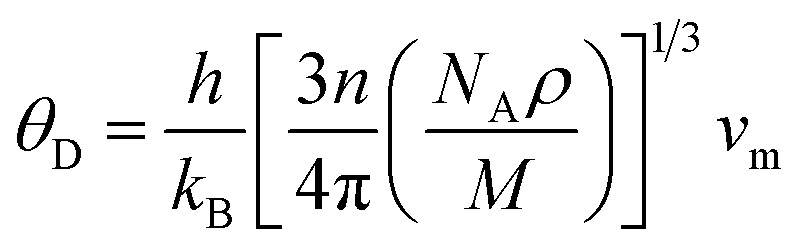
where *ℏ* represents the Planck's constant, *k*_B_ denotes the Boltzmann constant, *N*_A_ refers to Avogadro's number, *ρ* indicates the density, *M* signifies the molecular weight, and *v*_m_ stands for the mean sound velocity, which is:7
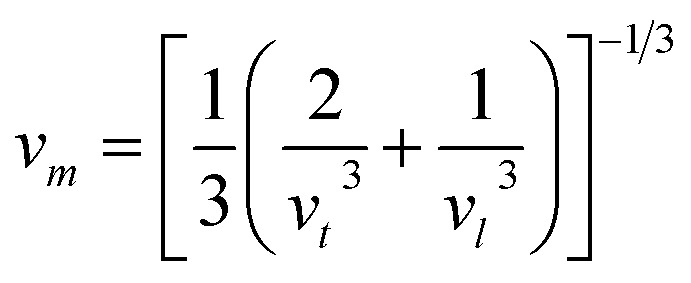
where *v*_l_ and *v*_t_ denote the longitudinal and transverse sound velocities, respectively, defined as:8
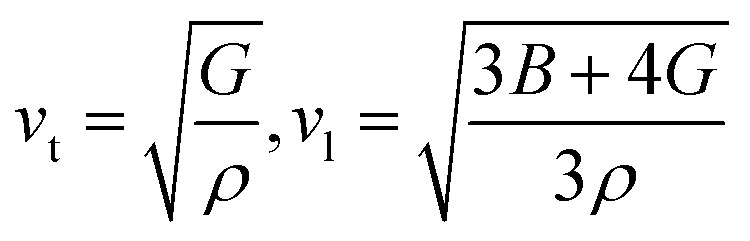


**Fig. 10:**
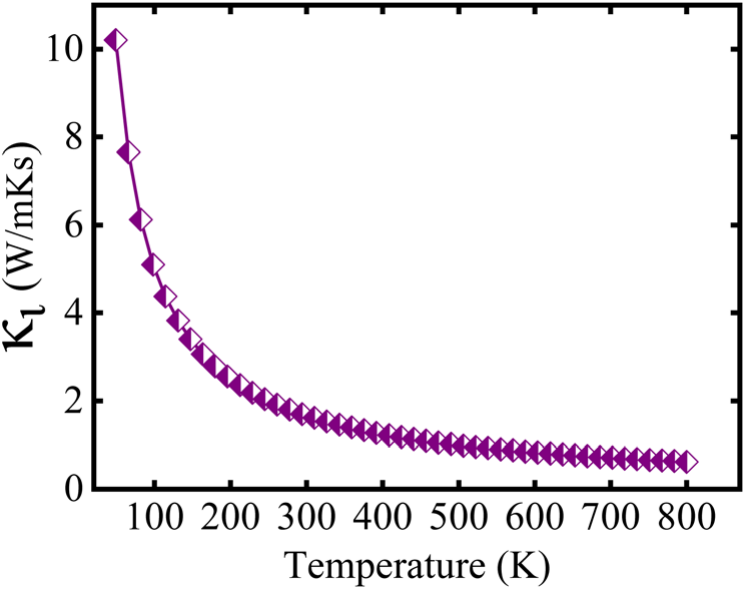
Computed lattice thermal conductivity (*k*_l_) as a function of temperature of the Lu_2_CoCrO_6_ structure.

The analysis presented in Fig. 10 describes that the calculated *κ*_*l*_ decreases as temp. increases, which is an advantageous outcome for TE applications. The observed reduction in *κ*_*l*_ corresponds to increased phonon scattering at higher temp., resulting from increased lattice vibrations that decrease the phonon mean free path. The measured value of *κ*_*l*_ is 1.66/1.00/0.71 Wm^−1^ K^−1^s at 300/500/700 K, which has an impact on the *ZT* value at elevated temp.

**Fig. 11:**
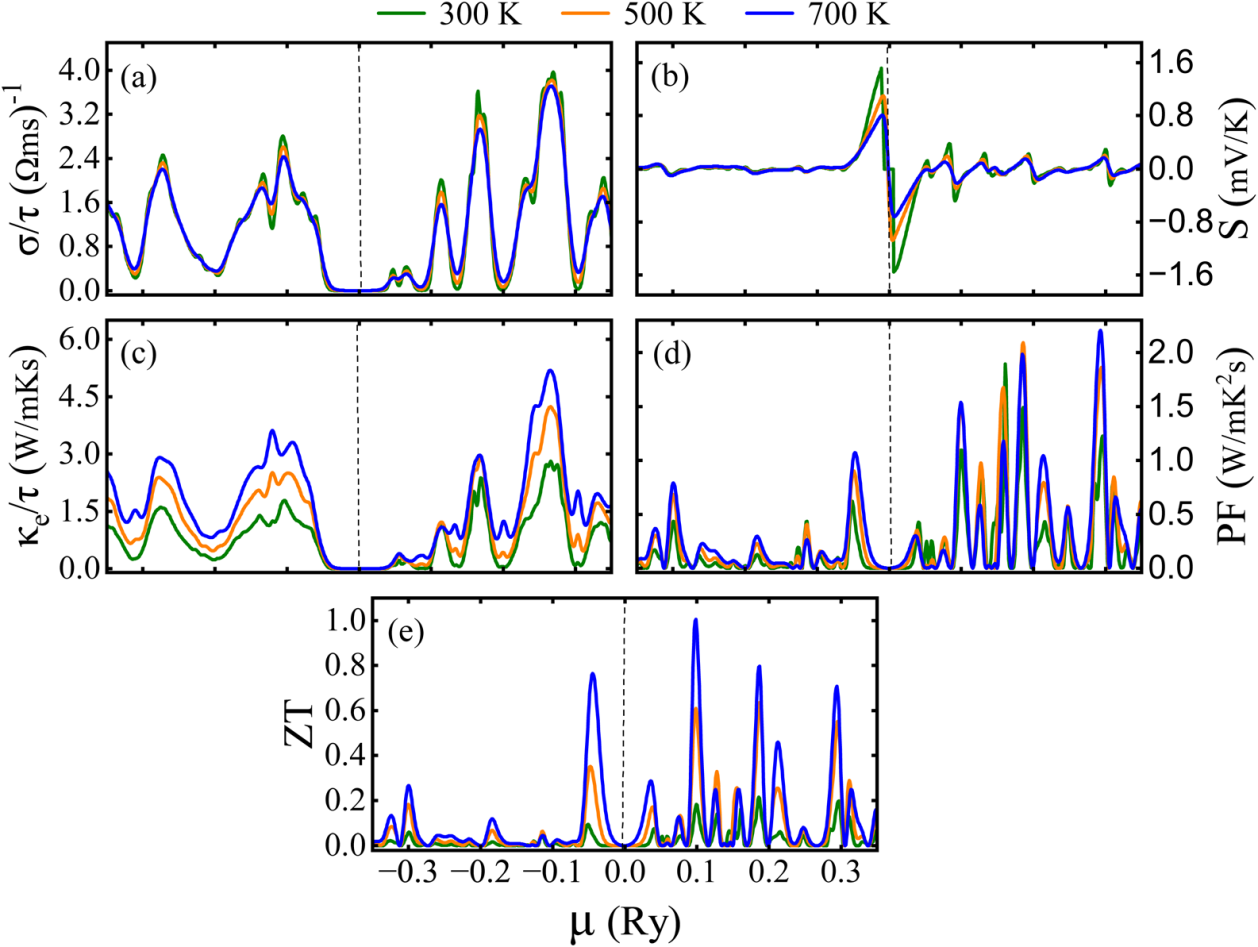
Computed numerous thermoelectric features as a function of chemical potential (*µ*) of the Lu_2_CoCrO_6_ structure.

Furthermore, the semi-classical Boltzmann transport theory, which is implemented in the BoltzTraP2 code,^61^ was used to study the transport aspects of the LCCO in the rigid band approximation under the constant scattering time approximation. The evaluation of TE parameters like electrical conductivity per relaxation time 
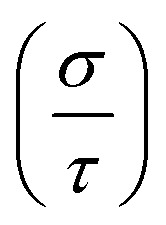
, S, electronic *κ* per relaxation time 
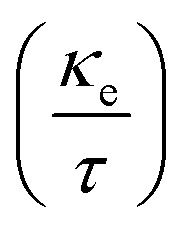
, 
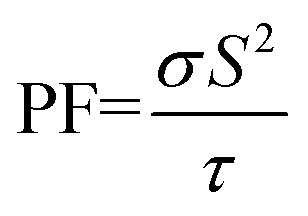
, and 
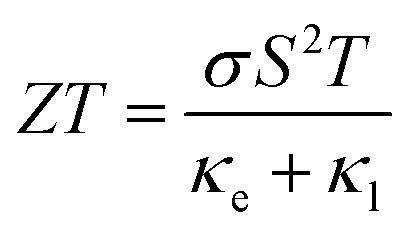
 was conducted as a function of the chemical potential (*µ*) within the range of −3.5 to 3.5 Ry. In this context, positive values of *µ* indicate n-type carriers, while negative values correspond to p-type ones. The computed 
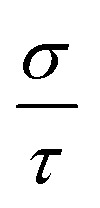
 is shown in Fig. 11, demonstrating minimal variations with temp. The peak exhibits at 300 K having a value of 3.9 × 10^19^ (Ω ms)^−1^ in the n-type region and 2.7 × 10^19^ (Ω ms)^−1^ for the p-type coincide with the CB edge, indicating that the majority of the n-type carrier concentration. The S depends on the contributions of both n and p-type charge carriers. As shown in Fig. 11(b), S shows positive values for p-type carriers (*E* − *E*_F_ < 0), having the highest value of 1.151 mV/K and negative values for n-type carriers (E − *E*_F_ > 0) with 1.153 mV K^−1^ at 300 K, where a sign change at the *E*_F_ signifying the transition between two conduction types. For n-type carriers, S reaches notably high absolute values at energies exceeding the *E*_F_. The magnitude of S typically decreases with temp. in both carrier types with in the given temp. range of 300–700 K. As predicted, 
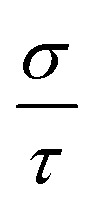
 rises significantly when *µ* moves into the conduction band (*µ* > 0), while *S* shows a peak close to the band edge at *µ* = 0.01 Ry and subsequently declines as *µ* increases. The inverse relationship between 
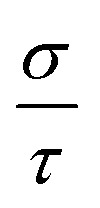
 and *S* is a defining feature of the TE materials, resulting from the opposing dependence of these features on the carrier concentration.

Next, the 
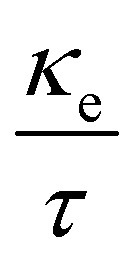
 exhibits a similar qualitative trend to 
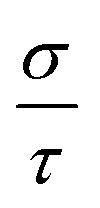
, showing maxima in areas of high carrier concentration and demonstrating moderate temp. dependence. The increase in 
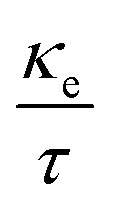
 with rising temp. is linked to the simultaneous enhancement in carrier mobility. It has the highest value of 5.19 × 10^14^ W mK^−1^ s^−1^ for n-type and 3.58 × 10^14^ W mK^−1^ s^−1^ for p-type region at 700 K, indicating n-type majority charge carriers. Now, the temp. dependence of the PF has been examined, revealing a linear increase in its value throughout the analyzed temp. range as displayed in Fig. 11(d). The peak is noted at 700 K owing the value of 2.20 × 10^11^ W mK^−2^ s^−1^ in the n-type and 1.06 × 10^11^ W mK^−2^ s^−1^ in the p-type region, indicating that LCCO attains enhanced TE performance at higher temp. Ultimately, the dimensionless *ZT* (see Fig. 11(e)), aligns with the trend of the PF while also being affected by the overall *κ*_e_ + *κ*_l_. In both p and n-type regimes, *ZT* shows notable peaks near the band edges, aligning with the maxima in the PF. The magnitude of *ZT* increases from 300 to 700 K primarily because the *κ*_e_ rises with temperature and the lattice thermal conductivity (*κ*_l_) decreases due to enhanced phonon–phonon scattering. Although *S* decreases at higher temperatures, the combined effect of improved charge carrier transport and suppressed *κ*_l_ results in a net enhancement of *ZT*.

## 4 Conclusion

In summary, thermodynamic, mechanical, dynamical, electronic, magnetic, and thermoelectric (TE) aspects of the Lu_2_CoCrO_6_ structure were analyzed using ab*initio* calculations. The energetic stability is confirmed by the computed negative formation enthalpy, and mechanical robustness is validated by the elastic constants that meet the Born criteria. Likewise, the dynamical stability of the system is confirmed by the phonon dispersion analysis, which reveals that no imaginary frequency modes across the Brillouin zone exist. Furthermore, the computed electronic structure calculations revealed that the system is a semiconductor having a direct band gap of 1.13 eV. Additionally, anti-ferromagnetic superexchange coupling is observed between Co^+3^ 3d^6^ and Cr^+3^ 3d^3^ ions *via* oxygen, confirms the ferrimagnetic spin ordering. This further affirms the 3D spin-magnitazion density plot and also the spin moments of Co (3.11 *µ*_B_) and Cr (−2.52 *µ*_B_) ions are align antiparallel. Also, the TE parameters varies as a function of chemical potential, such as low thermal conductivity, improved electrical conductivity, and a large Seebeck coefficient shows promising performance. Interestingly, a maximum figure of merit of 1.00 at a chemical potential of 0.1 eV under 700 K enhances its potential for TE application along with spintronics.

## Author contributions

Samia Shahzadi: writing – original draft, investigations, formal analysis, data curation. A. Elfasakhany: research support and resources. S. Nazir: writing – review and editing, validation, supervision, project administration, conceptualization.

## Conflicts of interest

The authors declare no competing interests.

## Supplementary Material

RA-015-D5RA07005H-s001

## Data Availability

The datasets used and/or analyzed during the current study are available from the corresponding author on reasonable request. Supplementary information (SI) is available. See DOI: https://doi.org/10.1039/d5ra07005h.

## References

[cit1] Basavarajappa M. G., Chakraborty S. (2022). ACS Mater. Au.

[cit2] Alam M., Ghosh L., Majumder S., Singh P., Kumar S. V., Dixit S., Kumar D., Anand K., Kumari S., Ghosh A. (2022). et al.. J. Phys. D: Appl. Phys..

[cit3] Cheong S.-W., Mostovoy M. (2007). Nat. Mater..

[cit4] Fiebig M., Lottermoser T., Fröhlich D., Goltsev A. V., Pisarev R. V. (2002). Nature.

[cit5] Chen X., Xu J., Xu Y., Luo F., Du Y. (2019). Inorg. Chem. Front..

[cit6] Mandal T. K., Felser C., Greenblatt M., Kübler J. (2008). Phys. Rev. B: Condens. Matter Mater. Phys..

[cit7] Feng H. L., Arai M., Matsushita Y., Tsujimoto Y., Guo Y., Sathish C. I., Wang X., Yuan Y.-H., Tanaka M., Yamaura K. (2014). J. Am. Chem. Soc..

[cit8] Kobayashi K.-I., Kimura T., Sawada H., Terakura K., Tokura Y. (1998). Nature.

[cit9] Qian Y., Wu H., Kan E., Lu J., Lu R., Liu Y., Tan W., Xiao C., Deng K. (2013). J. Appl. Phys..

[cit10] Iliev M., Padhan P., Gupta A. (2008). Phys. Rev. B: Condens. Matter Mater. Phys..

[cit11] Yin W.-J., Weng B., Ge J., Sun Q., Li Z., Yan Y. (2019). Energy Environ. Sci..

[cit12] Lin C., Zhao Y., Liu Y., Zhang W., Shao C., Yang Z. (2021). J. Mater. Res. Technol..

[cit13] Mohassel R., Amiri M., Abbas A. K., Sobhani A., Ashrafi M., Moayedi H., Salavati-Niasari M. (2020). J. Mater. Res. Technol..

[cit14] Murthy J. K., Chandrasekhar K. D., Murugavel S., Venimadhav A. (2015). J. Mater. Chem. C.

[cit15] Anshul A., Kotnala R. K., Aloysius R. P., Gupta A., Basheed G. A. (2014). J. Appl. Phys..

[cit16] Rathi A., Borkar H., Rout P., Gupta A., Singh H., Kumar A., Gahtori B., Pant R., Basheed G. (2017). J. Phys. D: Appl. Phys..

[cit17] Dass R., Goodenough J. (2003). Phys. Rev. B: Condens. Matter Mater. Phys..

[cit18] Nasir M., Khan M., Kumar S., Bhatt S., Patra N., Bhattacharya D., Jha S. N., Biring S., Sen S. (2019). J. Magn. Magn. Mater..

[cit19] Sazonov A., Troyanchuk I., Sikolenko V., Szymczak H., Bärner K. (2007). Phys. Status Solidi B.

[cit20] Das R. R., Lekshmi P. N., Das S., Santhosh P. (2019). J. Alloys Compd..

[cit21] Kumar D., Kumar S., Sathe V. G. (2014). Solid State Commun..

[cit22] Yang W., Liu X., Zhao H., Chen X. (2014). J. Magn. Magn. Mater..

[cit23] Rathi A., Borkar H., Rout P., Gupta A., Singh H., Kumar A., Gahtori B., Pant R., Basheed G. (2017). J. Phys. D: Appl. Phys..

[cit24] Wu H., Pei Z., Xia W., Lu Y., Leng K., Zhu X. (2020). J. Alloys Compd..

[cit25] Nechache R., Harnagea C., Li S., Cardenas L., Huang W., Chakrabartty J., Rosei F. (2015). Nat. Photon..

[cit26] Gaikwad V. M., Brahma M., Borah R., Ravi S. (2019). J. Solid State Chem..

[cit27] Patterson F. K., Moeller C. W., Ward R. (1963). Inorg. Chem..

[cit28] Vasala S., Karppinen M. (2015). Prog. Solid State Chem..

[cit29] Murtaza G., AlObaid A. A., Al-Muhimeed T. I., Al-Qaisi S., Rehman A., Hegazy H., Nazir G., Morsi M., Mahmood Q. (2021). et al.. Chem. Phys..

[cit30] Wu T., Gao P. (2018). Materials.

[cit31] Mir S. A., Gupta D. C. (2019). Int. J. Energy Res..

[cit32] Ullah M., Khan S. A., Murtaza G., Khenata R., Ullah N., Omran S. B. (2015). J. Magn. Magn. Mater..

[cit33] Maiti T., Saxena M., Roy P. (2019). J. Mater. Res..

[cit34] Wu H., Shi X.-L., Liu W.-D., Li M., Gao H., Zhou W., Shao Z., Wang Y., Liu Q., Chen Z.-G. (2021). Chem.–Eng. J..

[cit35] Aswal D. K., Basu R., Singh A. (2016). Energy Convers. Manag..

[cit36] Mingo N. (2004). Appl. Phys. Lett..

[cit37] Toberer E. S., Zevalkink A., Snyder G. J. (2011). J. Mater. Chem..

[cit38] Mustafa G. M., Slam A., Saba S., Noor N., Iqbal M. W., Dahshan A. (2023). Polyhedron.

[cit39] Blaha P., Schwarz K., Sorantin P., Trickey S. (1990). Comput. Phys. Commun..

[cit40] Calderon C. E., Plata J. J., Toher C., Oses C., Levy O., Fornari M., Natan A., Mehl M. J., Hart G., Nardelli M. B. (2015). et al.. Comput. Mater. Sci..

[cit41] Madsen G., Singh D. J. (2006). Comput. Phys. Commun..

[cit42] Chatterjee S., Dutta A., Das I. (2024). J. Appl. Phys..

[cit43] Corrêa H., Cavalcante I., Souza D., Santos E., Orlando M. D., Belich H., Silva F., Medeiro E., Pires J., Passamai J. (2010). et al.. Cerâmica.

[cit44] Constable E. C., Catherine E., Schaffner S., Shardlow E. (2006). Acta Cryst..

[cit45] Serrate D., De Teresa J., Ibarra M. (2006). J. Phys. Condens. Matter..

[cit46] Wu X., Vanderbilt D., Hamann D. R. (2005). Phys. Rev. B: Condens. Matter Mater. Phys..

[cit47] Shang S., Wang Y., Liu Z.-K. (2007). Appl. Phys. Lett..

[cit48] Mouhat F., Coudert F.-X. (2014). Phys. Rev. B: Condens. Matter Mater. Phys..

[cit49] Priyambada A., Mohanty A., Parida P. (2023). Mater. Today Commun..

[cit50] Pugh S. F. (1954). Philos. Mag..

[cit51] Surucu G. (2018). Mater. Chem. Phys..

[cit52] Gencer A., Surucu G. (2018). Mater. Res. Express.

[cit53] Pan Y., Guan W. M., Li Y. Q. (2018). Phys. Chem. Chem. Phys..

[cit54] Quan S., Liu C., Jiang W., Zhang X. (2019). Phys. Rev. B.

[cit55] Lv Y., Zhang X., Jiang W. (2018). Ceram. Int..

[cit56] Otero-De-La-Roza A., Abbasi-Pérez D., Luaña V. (2011). Comput. Phys. Commun..

[cit57] Otero-de-la Roza A., Luaña V. (2011). Phys. Rev. B: Condens. Matter Mater. Phys..

[cit58] Wei J., Guo Y., Wang G. (2023). RSC Adv..

[cit59] Sofi S. A., Gupta D. C. (2021). Int. J. Energy Res..

[cit60] Anderson O. L. (1963). J. Phys. Chem. Solids.

[cit61] Madsen G. K., Carrete J., Verstraete M. J. (2018). Comput. Phys. Commun..

