# Supplementary material for: High structural stability, reduced lattice-thermal conductivity, and elevated energy harvesting efficiency in a Lu2CoCrO6
Source: RSC Adv. 2025 Nov 24;15(54):45986–95. doi: 10.1039/d5ra07005h (PMC12642989; doi:10.1039/d5ra07005h)
Supplement: RA-015-D5RA07005H-s001 [file RA-015-D5RA07005H-s001.pdf]

Supplementary Information (SI) for RSC Advances.
This journal is © The Royal Society of Chemistry 2025

Supporting Information

High Structural Stability, Reduced Lattice-Thermal Conductivity, and Elevated Energy Harvesting Efficiency in a Lu_2CoCrO_6

Samia Shahzadi[1], A. Elfasakhany[2], and S. Nazir[1*]

[1] *Department of Physics, University of Sargodha, 40100 Sargodha, Pakistan and*
[2] *Mechanical Engineering Department, College of Engineering, Taif University, Taif 21944, Saudi Arabia*

[*] Electronic address: safdar.nazir@uos.edu.pk,Tel:$+$92-334-971-9060

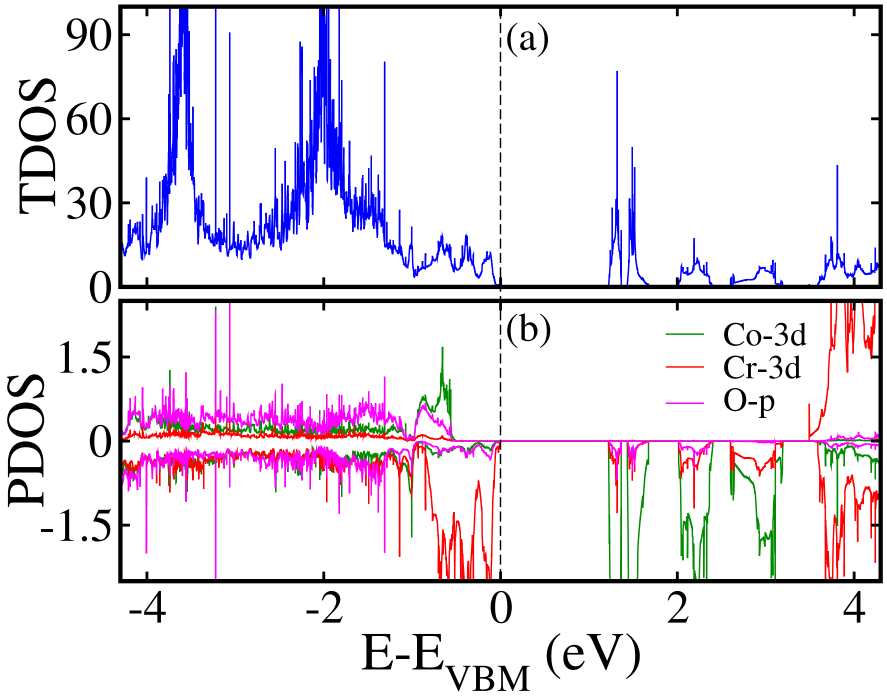

FIG. 1S: (Color line) GGA+U+SOC calculated (a) total density of states (TDOS) and (b) partial density of states (PDOS), highlighting the role of Co-3d/Cr-3d/O-2p orbitals around the Fermi level for the Lu_2CoCrO_6 structure.
